# Perianal Linear Syringocystadenoma Papilliferum: A Case Report with Review of the Literature

**DOI:** 10.30699/IJP.2023.1999659.3094

**Published:** 2023-10-15

**Authors:** Naser Tayyebi Meibodi, Yalda Nahidi, Mostafa Izanlu, Negin Davoodi, Sima Davoodi

**Affiliations:** 1 *Cutaneous Leishmaniosis Research Center, Mashhad University of Medical Sciences, Mashhad, Iran*; 2 *Department of Pathology, Imam Reza Hospital, Mashhad University of Medical Sciences, Mashhad, Iran*; 3 *Student Research Committee, School of Medicine, Shahrood University of Medical Sciences, Shahrood, Iran*

**Keywords:** Adnexal tumor, Apocrine gland, Eccrine gland, Linear, Syringocystadenoma papilliferum

## Abstract

Syringocystadenoma papilliferum is a rare benign adnexal tumor that originates from the apocrine and eccrine glands. It mainly manifests as a solitary lesion in the head and neck; however, rarely, it may involve the trunk and limbs, typically with a linear pattern. Here, we report an extremely rare case of congenital linear syringocystadenoma papilliferum on the left buttock near the anus in a 6-year-old girl. This lesion should be considered in the list of differential diagnosis of linear lesions in order to prevent complications with proper diagnosis, treatment or follow-up.

## Introduction

The syringocystadenoma papilliferum (SCAP) is a benign adnexal neoplasm arising from the apocrine and eccrine glands (1). It is a relatively rare neoplasm that develops in 50% of cases at birth and in 15-30% of cases at puberty (2). In most cases, the SCAP manifests as solitary lesions in the head and neck. Simultaneous presence of the multiple lesions in a linear pattern outside head and neck region is an exceptional occurrence (3, 4). In 30% of the cases, the SCAP may arise from a nevus sebaceous (5). In this case report, we present a case of the congenital linear SCAP in the left buttock of a 6-year-old girl.

## Case Presentation

**Fig. 1:**
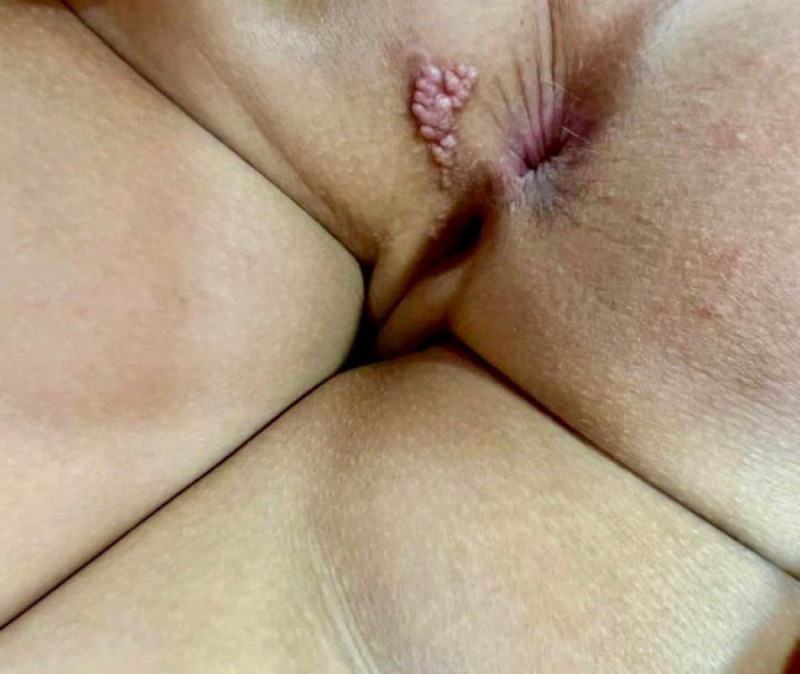
Multiple soft pink pseudovesicular papules with a linear distribution on the left buttock near the anus

The patient was a healthy 6-year-old girl who presented with slowly growing multiple soft pink pseudovesicular papules with a linear distribution on the left buttock near the anus (Figure 1). According to the patient's parents, the lesions have been mung-sized pink papules, which existed since birth, but over time, especially during the last year, their size and number had been increased. During this period, the lesions were asymptomatic. 

The patient's vital signs were stable and through examination and medical history of the patient, no pathological or abnormal points, including neurological, ocular, skeletal or other skin lesions that may be present in the syndromes such as epidermal nevus syndrome were found. In addition, the patient had no history of taking any special medicine. Her family history was unremarkable. No special laboratory test or imaging was done for the patient.

**Fig. 2:**
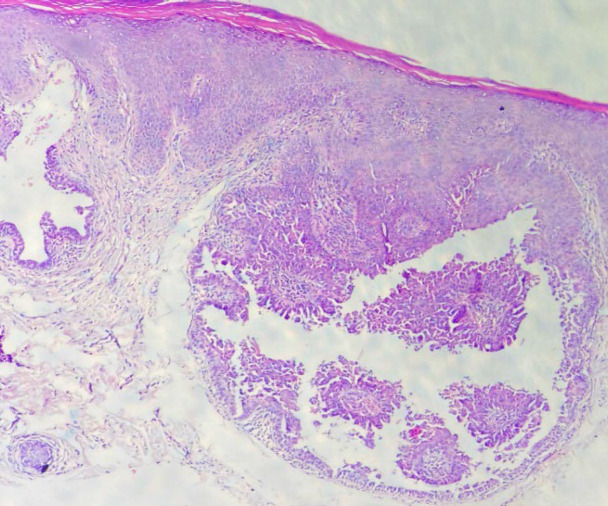
Cystic invaginations extending from the epidermis down to the dermis. The cystic structures were lined by papillae which had two layers of columnar epithelium with decapitation secretion

**Fig. 3. A, B:**
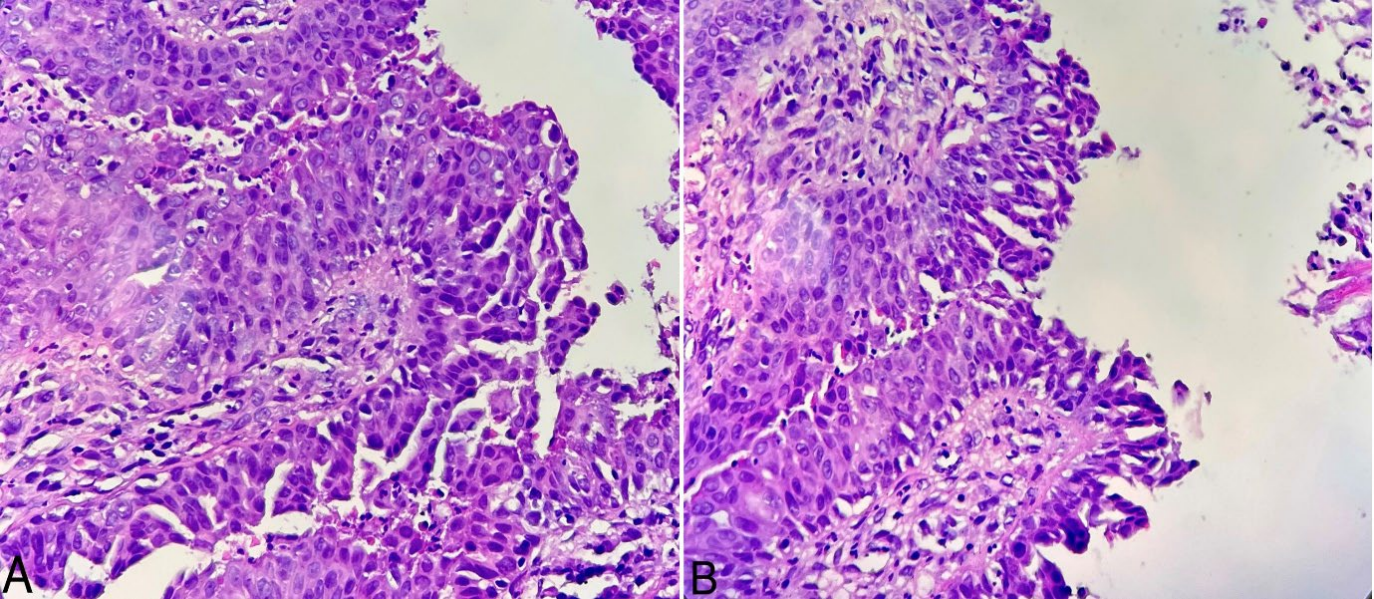
The epithelium showed double layers of cells consisting of an outer layer of cuboidal cells and an inner luminal layer of tall columnar cells. Decapitation secretion was observed in the luminal layer. The fibrous stromal core showed plasma cell infiltration

Skin biopsy was obtained from one of the lesions in order to distinguish between several differential diagnoses such as epidermal nevus, lymphangioma circumscriptum, SCAP, wart, and molluscum contagiosum. Histopathologic examination revealed cystic structures lined by the papillae with two layers of cells. The inner layer consisted of tall columnar cells with apocrine cells and outer layer consisted of cubic cells. Decapitation secretion was also noted. Abundant edema and plasma cells existed in the stroma of papillary projects (Figures 2 and 3).

Finally, according to the clinical and histological manifestations, a diagnosis of the linear SCAP was made and the patient was referred to the Pediatric Surgery Department for the surgical removal of the lesion. There was no sign of recurrence in the 1- and 6-months follow-up appointments.

## Discussion

The SCAP is a benign cutaneous hamartoma originating from the apocrine and eccrine glands (6). It can develop either de novo or on a sebaceous nevus (7). In most cases, the lesions increase in size slowly, but in a number of patients, their size can increase significantly in a short period of time such as during puberty or pregnancy (8, 9). The lesions can be large, verrucous, ulcerated, crusted, and with serous fluid drainage (9).

There are three clinical forms for the SCAP including: A: plaque-type form, that can be seen as an alloptic plaque or patch on the patient's scalp and often increases in size around puberty and becomes verrucous. B: linear type, in which the lesions are often seen in the form of multiple papules in a linear pattern and predominant in the head and neck area. C: solitary nodular type that is often seen as a pedunculated nodule in the trunk, axilla, and shoulder (2). In most cases, the SCAP is seen as solitary lesions and the linear form is rare (10). Almost, 20 cases of its linear form have been previously reported in the literature (2).

Seventy-five percent of the reported cases of SCAP have been in the scalp, 20% of the cases have been in the trunk, abdomen, flank, and only 5% of the cases have been in the limbs (1). Rarely, it has also been reported in the groin, buttocks, and anogenital area (7).

The localization of SCAP over the anogenital area and buttocks has been documented in sixteen cases in the previous studies. Among them, only 4 cases have been reported in the perianal area (7).

The SCAP can be seen in association with other benign and malignant tumors such as nevus sebaceous, apocrine adenoma, ductal eccrine carcinoma, basal cell carcinoma, and trichoepithelioma (1). Malignant transformation to the basal cell carcinoma, squamous cell carcinoma, or sweat gland carcinoma has been reported in plaque, solitary, and nodular forms, but this transformation has not been described in the linear form, possibly due to the de novo nature of this form (3).

For the linear form of SCAP, differential diagnoses such as epidermal nevus, nevus sebaceous, basaloid follicular hamartoma, eccrine nevus, wart, pyogenic granuloma, subcutaneous fungal infection, and giant lymphangioma can be considered and Histopatholog examination can confirm the final diagnosis(4). In children, viral diseases such as wart and molluscum contagiosum, as well as other adnexal tumors may be included in the differential diagnosis of SCAP (9).

In the histopathological examination of SCAP, cystic spaces and papillary structures are observed. The papillae protrude towards the cystic cavities. The wall of cystic cavities consists of two layers of cells: long columnar cells inside and cubic cells outside (1). Decapitation secretion is also often seen on the surface of the lumina (4). Edema and plasma cells are often noted in the stroma of these structures (1, 4).

Although staining of the luminal cells with alcian blue, colloidal iron, and diastase resistant Periodic Acid-Schiff (PAS), as well as positive immunohistochemical staining for GCDFP-15, CD15, CEA and EMA reinforces the existence of apocrine differentiation in this tumor, some studies have reported eccrine differentiation to be the origin of this tumor cells (4).

Treatment options for the SCAP include surgical removal, Moh's microscopic surgery, or CO_2_ laser removal for the areas that are not appropriate for surgery (1, 3, 6, 9). In the present case, the patient was referred to the Pediatric Surgery Clinic and the lesion was removed surgically.

## Conclusion

The SCAP, which is a rare cutaneous hamartoma and is often seen as a single lesion on the scalp, can exceptionally be seen as multiple linear lesions in other areas of the body. In children close to puberty, there is a possibility of increasing size and becoming verrucous, ulcerous or crusting and causing cosmetic problems. In adults there is also a possibility of malignant transformation. Therefore, this lesion should be considered in the list of differential diagnosis of linear lesions in order to prevent complications with proper t diagnosis, treatment or follow-up.

## Funding

None.

## Conflict of Interest

The authors declared no conflicts of interest.
